# Mortality Rate and Ten Years Survival of Elderly Patients Treated with Total Hip Arthroplasty for Femoral Neck Fractures

**DOI:** 10.5704/MOJ.2107.020

**Published:** 2021-07

**Authors:** NS Nanchappan, S Chopra, A Samuel, L Therumurtei, SS Ganapathy

**Affiliations:** 1 Department of Orthopaedics, Hospital Sultanah Bahiyah, Alor Setar, Malaysia; 2 Institute for Public Health, National Institutes of Health, Shah Alam, Malaysia

**Keywords:** total hip arthroplasty (THA), surgical timing, mortality, survival analysis

## Abstract

**Introduction:**

Mortality following traumatic femoral neck fractures in the elderly (age >60 years) is influenced by many factors. Addressing some of them may reduce the mortality rate thus improving patient survival and quality of life.

**Materials and methods:**

This study was a retrospective research using data collected from Hospital Sultanah Bahiyah, Kedah between the years 2008-2018. We measured outcomes such as age, gender, hospital stay, default rate, ambulation post-surgery, American Society of Anaesthesiologists score (ASA) and surgical timing in correlation with mortality rate and 10-year survival of elderly patients treated with Total Hip Arthroplasty for femoral neck fractures in this centre.

**Results:**

A total of 291 traumatic femoral neck fractures aged above 60 years post total hip arthroplasty performed were included. There was higher number of female (n =233) compared to male (n=53) Estimated 10 years survival from Kaplan Meier was 42.88% (95% CI: 33.15, 52.54). One year mortality rate in our study was found to be 18.9%. The average time to event was 7.1 years (95% CI:33.15, 52.24) with a mean age group of 75.

**Discussion:**

Total hip arthroplasty patients not ambulating after surgery had a 4.2 times higher hazard ratio compared to ambulators. Those with pre-existing systemic disease (ASA III and IV) were found to have the highest hazard ratio, almost five times that of healthy patients, after adjusting for confounding factors. Delay of more than seven days to surgery was found to be a significant factor in 10-year survival with a hazard ratio of 3.8, compared to surgery performed earlier.

**Conclusion:**

Delay of more than 7 days to surgery in 10 years survival was significant with high hazard ratio. It is a predictor factor for survival in 10 years. A larger sample size with a prospective design is required to confirm our findings regarding “unacceptable surgical timing” for femoral neck fractures in patients above 60 years of age.

## Introduction

Total hip arthroplasty (THA) has become an accepted surgical intervention for femoral neck fractures in the elderly, showing lower long term mortality risk and better function compared to other forms of treatment^1^. In the late ’90s, the incidence of hip fracture in patients above 50 years of age in Malaysia was 90 per 100000 individuals^2^; this figure is increasing over the years. The ideal waiting time for surgery following a hip fracture has been quoted as 24 to 48 hours according to Lee DJ^3^; however, there are not many studies analysing unacceptable delay for hip fractures^4^. These cases are managed through a multidisciplinary approach. Patients with hip fractures in Malaysia have a mortality rate of up to 20% in the first year and only 25% will resume daily activities^5^. Global hip fracture mortality at one year is an estimated 22% (ASIA 17.89%, United States 21%, Europe 23.4% and Australia 24.9%6. Many factors influence mortality and morbidity in patients with hip fractures such as underlying illness, post-operative care, and financial aid for surgery.

In this study, we evaluated age, gender, hospital stay, default rate, ambulation post-surgery, American Society of Anaesthesiologists score (ASA) and surgical timing. We investigated the mortality rate and 10 years survival of elderly patients treated with Total Hip Arthroplasty for femoral neck fractures. Based on our observation many factors contribute towards the outcomes of total hip replacement in the geriatric neck of femur fracture. The risk factors can be broadly classified into non-modifiable and modifiable factors. In this paper, we would like to highlight modifiable factors namely surgical timing and ambulation post-surgery as a critical factor in enhancing outcome in the geriatric neck of femur fracture in short term and long-term follow-up. The modifiable risk factor is of paramount importance to clinical practice and acts as a keystone in improving clinical outcome for patients.

## Materials and Methods

We conducted a retrospective, non-randomise study done in Hospital Sultanah Bahiyah, Alor Setar, Kedah, Malaysia (single institution) from 2008 to 2018. Sample size estimation was done using G*Power, which showed a minimum size of 270 in reference to Clayer and Bauze^5^. The inclusion criteria included all patients above 60 years of age who have done a THA. Patients who had a pathological fracture (other than osteoporosis), revision surgery and less than 60 years old were excluded. We also excluded other than total hip arthroplasty surgery. We aimed to determine the mortality rate and 10 years survival of elderly patients treated with Total Hip Arthroplasty for femoral neck fractures in our centre. Data was collected from the arthroplasty operation theatre register, case notes and operative notes. Dates of death were obtained from the National Registration Department, Ministry of Home Affairs. Surgical timing, ASA, post-operative ambulatory status (through phone calls for defaulters or clinic notes for those on follow-up) and follow-up compliance were among variables investigated. Surgical timing was defined as the time taken for patients to undergo surgical intervention from the time of fracture. Our data collection was done by one researcher to prevent bias. It was collected over six months.

We aimed to evaluate age, gender, hospital stay, default rate, ambulation post-surgery, American Society of Anaesthesiologists score (ASA) and surgical timing, and to investigate mortality rate and 10 years survival of elderly patients treated with Total Hip Arthroplasty for femoral neck fractures. All descriptive results were presented in frequency and percentage. The Chi-Square test was used to determine the association between short term mortality and surgical timing. In the analysis of patient survival at 10 years, the end event was all-cause mortality. Survival rate and estimated median survival times were calculated using the Kaplan–Meier method. Factors predictive of 10-year survival of patients were determined using Multiple Cox regression analysis. Results were expressed as the hazard ratio (HR) within 95% confidence interval (95% CI) and were two-sided. All statistical analysis was performed using Statistical Package for Social Science (SPSS) Ver 23.0 and Stata 13.0; p values of less than 0.05 were considered to be significant. This study was approved by the Medical Research and Ethics Committee, Ministry of Health Malaysia (NMRR-19-4127-51227) on 17-September-2020.

Demographic data was analysed by multivariate analysis. The mortality rate was compared using the Chi-Square test. Survival Rate and Median Survival Time which were the secondary objectives are measured using Kaplan-Meier and Log Rank Statistics. Predictive factors for 10-year Survival among THA patients were done using Multiple Cox Regression.

## Results

A total of 450 cases of THA were performed over the 10 years 2008-2018 for all causes inclusive of advanced avascular necrosis, osteoarthritis, neglected development dysplasia, revision surgery, and pathological fractures. Two hundred ninety-one cases satisfied the criteria and were included in this study.

The demographic and clinical characteristics of the patients is presented in Table I. The oldest patient was 98 years of age. Most patients were females (80.1%) and the majority (73.2%) had an ASA score of II (mild systemic disease). 73.9% waited more than seven days for surgery. A total of 89% of patients were walking with/without aid after surgery.

**Table I:** Demographic and clinical characteristics of THR patients (n=291)

Characteristic	Frequency	Percentage%
Age		
60 – 69	77	26.5
70 – 79	114	39.2
80 and above	100	34.4
Gender		
Male	58	19.9
Female	233	80.1
ASA		
Healthy	36	12.4
Mild	213	73.2
Severe/cons	42	14.4
Time for Op (days)		
3 and less	29	10.0
4 – 7	47	16.2
Above 7	215	73.9
Hospital Stay (days) (including pre-operative)		
1 – 3	72	24.7
4 – 7	142	48.8
8 – 10	19	6.5
Defaulted follow-up (subsequently traced)		
Yes	188	64.6
No	103	35.4
Ambulation		
Yes	259	89.0
No	32	11.0

Table II presents the overall 10-year survival rate and median survival time by demographic and clinical characteristics. Females had a higher survival rate compared to males (48.4% vs 28.5%). Survival rate was also seen to decrease with increasing duration of hospital stay, ASA category and time to surgery. Patients who defaulted follow-up and those not ambulating were found to have significantly lower survival rate and median survival time compared to their counterparts.

**Table II:** 10-year survival rate and median survival time by demographics and clinical characteristics (Kaplan-Meier and log rank statistics)

Variables	% Survival Rate (95% CI)	Median Survival time (95% CI)	Log rank Statistic	p value
Overall	43.9 (33.8 – 53.6)	84.7 (62.9 – 106.5)		
Age				
60 – 69	60.1 (32.3 – 79.5)	-		
70 – 79	50.3 (35.2 – 63.6)	-	2.949	0.086
80 and above	22.6 (9.5 – 39.0)	84.7 (62.9 – 106.5)	15.404	<0.001
Gender				
Male	28.5 (13.8 – 45.0)	53.9 (21.3 – 86.5)	11.036	0.001
Female	48.4 (36.4 – 59.4)	103.6		
Hospital Stay				
1 – 3 days	67.3 (48.9 – 80.3)	-		
4 – 7 days	47.4 (30.1 – 62.8)	101.7	0.006	0.939
8-10 days	33.5 (9.5 – 60.1)	74.5 (63.7 – 85.3)	0.213	0.644
> 10 days	31.8 (18.8 – 45.6)	50.0 (5.3 – 94.7)	8.570	0.003
Defaulted follow-up				
Yes	38.8 (28.3 – 49.1)	72.7 (63.6 – 81.8)	7.096	0.008
No	54.4 (29.5 – 73.9)	-		
Ambulation				
Yes	48.9 (37.7 – 59.2)	103.6	44.323	<0.001
No	8.7 (0.6 – 30.5)	6.1 (0.0 – 14.0)		
ASA				
Healthy	57.8 (33.0 – 76.2	-		
Mild (II)	48.4 (36.4 – 59.3)	92.7	0.983	0.322
Severe/cons				
(III and IV)	18.5 (5.8 – 36.8)	19.8 (8.6 – 31.0)	14.490	<0.001
Time for Op (days)				
3 and less	76.7 (50.5 – 90.2)	-		
4 – 7	62.6 (41.4 – 78.0)	-	0.941	0.332
Above 7	29.4 (15.7 – 44.6)	72.7 (58.9 – 86.5)	8.022	0.005

**Fig. 1:**
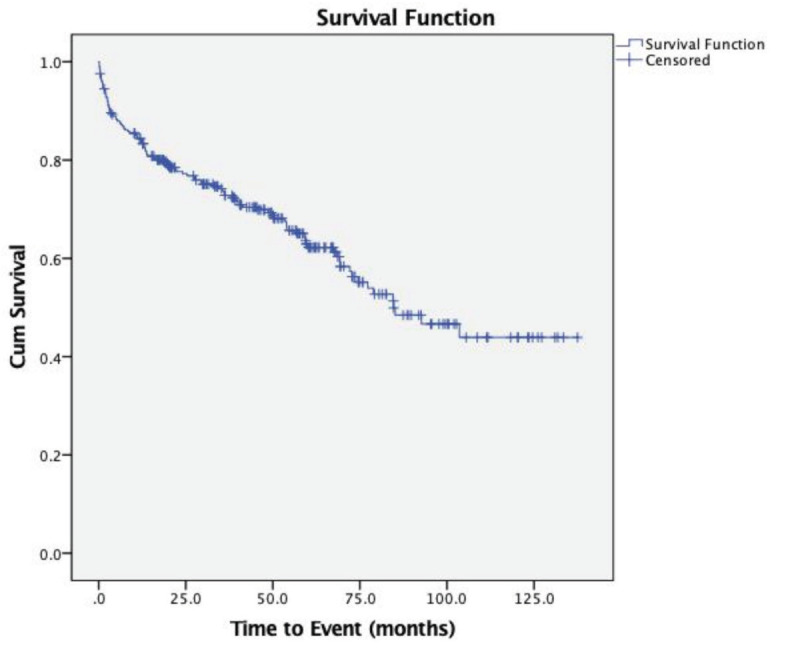
Kaplan Meier curve for overall survival estimates among THR patients (n=293).

The overall 10-year probability of an event for this study is shown in Fig. 1. The 10-year survival rate from Kaplan Meier estimated was 42.88% (95% CI: 33.15, 52.24). The median time for the overall event was 84.5 months (95% CI: 64.13, 104.88).

Table III, Multiple Cox Regression shows that patients of 80 years and above who underwent THA had almost three times a higher hazard ratio of mortality compared to those between the age of 60 to 69, after adjusting for confounders. Males were found to have a two times higher hazard ratio. Duration of hospital stay was found to be an insignificant factor for 10-year all-cause mortality. Total Hip Arthroplasty patients not ambulating after surgery had a 4.2 times higher hazard ratio. Those with pre-existing systemic disease (ASA III and IV) were found to have the highest hazard ratio, almost five times that of healthy patients after adjusting for confounding factors. A delay of more than seven days to surgery was also found to be a significant factor in the 10-year survival with a hazard ratio of 3.8, compared to surgery performed within three days. The results of multiple cox regression analysis are presented in Table IV.

**Table III:** Association between mortality and time for op (days) for THR patients (n=291)

	Time for Op (Days)		Chi Square test	
	3 and less	4 to 7	Above 7	p value
1 month mortality				
Alive	27	45	199	0.510
Passed Away	0	2	10	
3 month mortality				
Alive	27	44	184	0.099
Passed Away	0	3	25	
1 year mortality				
Alive	26	41	171	0.125
Passed Away	1	6	38	

**Table IV:** Predictive factors for 10-year Survival among THA patients using Multiple Cox Regression (n=291)

Variables	Regression Coefficient (b)	Adjusted HR (95% CI)	Wald Stats	p value
Age				
60 – 69		Ref		
70 – 79	0.61	1.85 (1.00, 3.42)	3.83	0.051
80 and above	1.12	3.05 (1.68, 5.54)	13.51	<0.001
Gender				
Male	0.70	2.01 (1.30, 3.13)	9.73	0.002
Female		Ref		
Hospital Stay				
1 – 3 days		Ref		
4 – 7 days	0.04	1.04 (0.58, 1.88)	0.02	0.890
8-10 days	-0.41	0.67 (0.28, 1.60)	0.82	0.364
> 10 days	0.57	1.77 (0.94, 3.31)	3.16	0.076
Defaulted follow-up				
Yes	0.42	1.52 (0.95, 2.42)	3.04	0.081
No (traced)		Ref		
Ambulation				
Yes		Ref		
No	1.43	4.19 (2.52, 7.00)	30.18	<0.001
ASA				
Healthy		Ref		
Mild	0.38	1.46 (0.74, 2.88)	1.16	0.282
Severe/cons	1.58	4.86 (2.25, 10.49)	16.21	<0.001
Time for Op (days)				
3 and less		Ref		
4 – 7	1.01	2.74 (0.94, 8.00)	3.39	0.066
Above 7	1.34	3.83 (1.48, 9.93)	7.62	0.006

Notes: - Backward LR and forward LR cox proportional hazard regression model applied. - Multicolinearity and two way interaction term checked. - Log-minus-log plot and hazard function plot wer

Table IV compares surgical timing and mortality at one month, three months and one year showed no significant association with p-value > 0.05. A 10-year median life span of fracture patients undergoing THA for all-cause mortality was found to be 84.7 months or 7.1 years (95% CI 62.9, 106.5). The survival rate at 10 years is 43.9% (95% CI 33.8– 53.6).

## Discussion

THA for femoral neck fractures is currently the generally accepted mode of treatment for elderly patients. One year mortality rate in our centre was 18.9% which was marginally less than the 20% reported by Lee *et al* in the Malaysian population^2^. The overall mortality rate at 1.3 and 12 months in our study was found to be 4.43%, 11% and 18.9%, respectively. Patients with early surgery (within three days) had lower mortality (0%, 0%, 3.34%); however, we could not demonstrate that this was significant on statistical analysis. In comparison a meta-analysis by Berstock *et al* (2014), showed mortality at one and three months following hip replacement to be 0.30% and 0.65%^7^; surgical timing was not stated. This was in THA being performed for hip fractures but excluded mortality among patients with diabetes, rheumatoid arthritis and extremes of age. Uzoigwe *et al* reported on 2056 patient operated within 12, 24 and after 36 hours^8^; there was increased mortality in patients operated after 36 hours^8^.

Surgical timing was found to be a significant predictive factor for 10-year survival in our study. Patients undergoing surgery within seven days had better survival at 10 years compared to those waiting longer. This contrasts with some studies which show mortality is higher if surgery is performed after 48 hours^8^. Our study appears to show that delay of up to seven days is not deleterious. Causes of delay for surgery are various including co-morbidities or financial reasons.

Optimising co-morbid disease contributes to delay in performing surgery. Certain hospitals are privileged to have ‘hip fracture units’, a multidiscipline team consist of geriatricians, orthopaedic surgeons, anaesthetists and rehabilitation physicians in managing fragility fractures. Walton et al showed a lower mortality rate among the elderly in such units; however further research is required to prove the benefits^9^.

Our centre is a tertiary hospital with many subspecialties. It is also a referral centre for the state and neighbouring state. Securing surgical slot is competitive and overwhelming due to patient volume, traffic, and congestion. This may arbitrarily lead to prolong waiting time exceeding a seven days. Some may develop symptomatic bronchopneumonia or urinary tract infection due to being bed-bound and this would inadvertently further increase surgical timing.

Another paramount factor is financial issues. The patient who cannot afford prostheses costs need to apply through a ‘National Medical Fund’. This involves certain procedures which can be completed as early as three days but can take up to two weeks. In some developed countries, prostheses are stationed in public hospital operating theatres, readily available to be used when needed.

In our study, the estimated 10 years survival rate by Kaplan Meier analysis was 43.9% (95% CI: 33.15, 52.54). The average time to death was 7.1 years (95% CI:33.15, 52.24) for a mean age group of 75. Ramiah *et al* (Swedish hip registry 1993-2004), reported time to death as 5.6 years for a mean age group of 6510. Survival at 10 years was 89% for patients less than 65 years, 75% between the age of 65 to 74 and 51% in patients over 7510. In contrast, the survival rate of patients following hip hemiarthroplasty in females above 70 years of age was 73% at 1 year, 23% at 5 years, and only 6% at 10 years reported by Wacht *et al* (2003)^11^. Lee *et al* compared mortality and survival analysis between hip fracture and cancer patients (thyroid, breast) at five years. They concluded hip fracture should not be overlooked as the results were comparable^12^.

Poor post-surgical mobilisation is another significant variable in our study that was found to contribute to an increase in mortality. As mentioned by Baer *et al*, mobilisation should be within 24 hours post-surgery^13^. However, this might not be possible as patients may still be under continuous epidural infusion. One advantage of performing THA compared to internal fixation in hip fractures is the ability to weight bear early after surgery^13^. We found in our study 32 patients were non-ambulators and all defaulted follow-ups. A total of 18 of them were bed-bound due to underlying illnesses such as stroke while the rest were untraceable and have been included as non-ambulators for this study (worst-case scenarios). Five out of this group of patients are still alive.

In our centre, patients who have undergone THA are followed-up long term at yearly intervals. Despite this, we found 35.4% had defaulted. After tracing, 14 could not be located due to demographic changes. Therefore, there were finally 4.8% that were unaccounted for.

Our study analysed mortality rate and survival for traumatic femoral neck fractures treated with THA. There is limited data available locally and internationally on patient survival at 10 years. Limitations of this study include a retrospective design, a relatively small number of patients and inability to trace some defaulted cases.

The magnitude of mortality rate in geriatric hip fractures has been discussed elaborately in the available literature. Non-modifiable risk factors investigated contributes to the outcome, morbidity, and mortality rate of the patients. This predisposes a patient to a higher risk of circulatory, pulmonary, infection and haemostatic failure thus leading to higher morbidity and mortality.

A silver lining in the cloud is to address the modifiable factors which are within the means of clinicians in enhancing the outcome post-surgery. The search of studies regarding Surgical timing and rehabilitation are modicum in comparison to other risk factors.

Hence, it is established via this retrospective observation, surgical timing plays an important role in the mortality rate of geriatric patients undergoing THR for NOF fracture. The significance is established concerning 10 years survival rate. However, it was not significant in the first three months and one year and this is incoherent with the possibility of other causes of mortality such as cardiorespiratory failures, venous thromboembolism, infection, pulmonology causes and many more which has been extensively elaborated in literature.

Surgical timing imposes its effect on long term outcome. This is salient information as we are long for a long-term survival rate for the geriatric community with a good quality of life. This is not a solitary factor for successful long-term outcome but works well if done in a multidisciplinary approach. Surgical timing will be an adjuvant pillar in deciding the long-term outcome for the patients. The factors that help in this outcome are postulated to be: quick surgery leading to quick rehabilitation, delaying the onset of sarcopaenia, restoring confidence to patients as it is done early, early commencement of anti-osteoporotic drug and quick return to the community.

There are several limitations to this retrospective study conducted, including the causes of death that were not discussed in detail.

## Conclusion

Non-ambulating patients post-surgery, ASA III and above as well as an age of more than 80 years were significant contributing factors for 10-year survival in our analysis. Surgical timing within seven days was not related to mortality at one month, three months and one year. Delay of more than seven days to surgery in 10-year survival was significant with high hazard ratio. It is a predictor factor for survival in 10 years. A larger sample size with a prospective design is required to confirm our findings regarding “unacceptable surgical timing” for femoral neck fractures in patients above 60 years of age.
